# Brain Tissue Volumes and Perfusion Change with the Number of Optic Neuritis Attacks in Relapsing Neuromyelitis Optica: A Voxel-Based Correlation Study

**DOI:** 10.1371/journal.pone.0066271

**Published:** 2013-06-18

**Authors:** Carlos A. Sánchez-Catasús, José Cabrera-Gomez, William Almaguer Melián, José Luis Giroud Benítez, Rafael Rodríguez Rojas, Jorge Bosch Bayard, Lídice Galán, Reinaldo Galvizu Sánchez, Nancy Pavón Fuentes, Pedro Valdes-Sosa

**Affiliations:** 1 Center for Neurological Restoration, Havana, Cuba; 2 Salvador Allende" Hospital, Havana, Cuba; 3 Cuban Neuroscience Center, Havana, Cuba; University of Jaén, Spain

## Abstract

Recent neuroimaging studies show that brain abnormalities in neuromyelitis optica (NMO) are more frequent than earlier described. Yet, more research considering multiple aspects of NMO is necessary to better understand these abnormalities. A clinical feature of relapsing NMO (RNMO) is that the incremental disability is attack-related. Therefore, association between the attack-related process and neuroimaging might be expected. On the other hand, the immunopathological analysis of NMO lesions has suggested that CNS microvasculature could be an early disease target, which could alter brain perfusion. Brain tissue volume changes accompanying perfusion alteration could also be expected throughout the attack-related process. The aim of this study was to investigate in RNMO patients, by voxel-based correlation analysis, the assumed associations between regional brain white (WMV) and grey matter volumes (GMV) and/or perfusion on one side, and the number of optic neuritis (ON) attacks, myelitis attacks and/or total attacks on the other side. For this purpose, high resolution T1-weighted MRI and perfusion SPECT imaging were obtained in 15 RNMO patients. The results showed negative regional correlations of WMV, GMV and perfusion with the number of ON attacks, involving important components of the visual system, which could be relevant for the comprehension of incremental visual disability in RNMO. We also found positive regional correlation of perfusion with the number of ON attacks, mostly overlapping the brain area where the WMV showed negative correlation. This provides evidence that brain microvasculature is an early disease target and suggests that perfusion alteration could be important in the development of brain structural abnormalities in RNMO.

## Introduction

Neuromyelitis optica (NMO or Devic's disease) is a severely disabling autoimmune inflammatory demyelinating disorder of the central nervous system (CNS), which is recognized today as a distinct clinical entity from multiple sclerosis (MS) (reviewed in [1]). NMO is also associated with serum antibodies (NMO-IgG) to aquaporin-4, the most abundant water channel in the CNS (reviewed in [2]).

NMO has traditionally been regarded as a disease without brain involvement. However, recent neuroimaging studies have shown that brain abnormalities are more frequent than earlier described [3]–[6]. Our group has previously reported brain abnormalities in NMO by standard magnetic resonance imaging (MRI) [7]. Other studies, using diffusion tensor (DT) MRI have found brain abnormalities in the normal appearing white matter (NAWM) [8]–[11]. Voxel-based morphometry studies have found white (WM) and grey matter (GM) atrophy [12], [13]. Investigations by functional MRI (fMRI), magnetization transfer (MT) MRI and DT MRI have also shown abnormalities in the normal appearing grey matter (NAGM) [8], [14]–[16]. Nevertheless, more research, considering multiple aspects of NMO, is necessary for a better understanding of the pathogenic mechanisms that cause brain abnormalities in this disease.

NMO is characterized by monophasic or recurrent attacks of optic neuritis (ON) and longitudinally extensive transverse myelitis (LETM) [17]. Unlike MS, a secondary progression course is uncommon in relapsing NMO (RNMO) [18]. A distinctive clinical feature of RNMO is that the incremental disability is attack-related. Consequently, attack-related changes might be expected on neuroimaging.

On the other hand, immunopathological analysis of NMO lesions revealed a unique vasculocentric pattern of complement activation [19]–[21], which has suggested that CNS blood microvessels could be early targets of the disease process. Therefore, brain perfusion could be altered. There are no previous studies confirming brain perfusion abnormalities in NMO. Brain tissue volume changes accompanying brain perfusion alteration could also be expected. Voxel-based correlation analyses using multiple neuroimaging modalities could help to identify brain tissue volumes and perfusion changes behind the attack-related process in patients with RNMO. Statistical parametric mapping (SPM) is well suited for this purpose [22].

The aim of this study is to investigate in RNMO patients whether there are associations between regional brain white (WMV) and grey matter volumes (GMV) and/or perfusion on one side, and the number of ON attacks, LETM attacks and/or total attacks on the other side. Because disease duration and NMO-IgG status could be also associated to brain structure and functional changes, we evaluate possible correlations of these two other clinical variables with regional brain tissue volumes and perfusion. Similar analyses are carried out with global tissue volumes. We demonstrate by SPM correlation analysis that regional brain WMV, GMV and perfusion change with the number of ON attacks in patients with RNMO.

## Materials and Methods

### Subjects

Twenty patients were initially enrolled as part of a research project in our institution. The patients were in stable phase of the disease (no acute relapse at least 4 months prior to the study) and all of them fulfilled Wingerchuk's revised diagnostic criteria for NMO: absolute criteria (ON and acute LETM) and the presence of at least two of the following three supportive criteria: (1) brain MRI negative or non diagnostic for MS at onset, (2) spinal cord MRI with contiguous T2-weighted signal abnormality extending over three or more vertebral segments, and (3) a serological test positive for NMO-IgG [17]. Five out of twenty patients were excluded from the study due to suboptimal quality of MRI or single photon emission computed tomography (SPECT) images or multiple or large brain WM lesions. The final group comprised 15 clinically confirmed RNMO patients. Nine of them were seropositive for NMO-IgG as demonstrated by an indirect immunofluorescence assay [23]. The characteristics of the participant patients (13 female, 2 male; mean age = 40.6±10.5 years; range = 17–56 years) are detailed in Table 1.

**Table 1:** Demographic and clinical characteristics of patients with relapsing neuromyelitis optica (n = 15).

**Sex (female/male)**	13/2
**Age (years)**	41 (17–56)
**Disease duration (years)**	8 (2–17)
**EDSS score**	5 (1–8.5)
**Total number of attacks**	5 (2–10)
**Number of ON attacks**	2 (1–4)
**Number of LETM attacks**	3 (1–8)
* **Interval from the last attack (years)**	2 (0.5–4)
* **Interval from the last ON attack (years)**	2.8 (0.5–4)
* **Interval from the last LETM attack (years)**	2 (0.5–4)
**Serological test for NMO IgG (positive/negative)**	9/6

Data shown as median and range (minimum, maximum). EDSS, Kurtzke Expanded Disability Status Scale [24]; ON, optic neuritis; LETM, longitudinally extensive transverse myelitis; NMO-IgG, antiaquaporin-4 autoantibody;

* Last attack MRI/SPECT time interval.

No macroscopic T1-visible lesions, including gadolinium enhanced lesions, were identified on brain MRI scans in any patient. Five patients had minor non-specific T2/FLAIR-visible lesions, three of which had less than five subcortical/deep WM lesions (less than 3 mm) and no juxtacortical localization, and another two patients had a single periventricular lesion without oval, ovoid or perpendicular orientation in morphology. The patients had not been treated with specific medications, such as immunosuppressants and corticosteroids, at least 4 months before the MRI and perfusion SPECT imaging.

As supplementary analyses, we also performed a group-comparison between NMO patients and control subjects for each image modality. The control group was composed of 15 clinically healthy volunteers (13 female, 2 male; mean age = 46.6±12.5 years; range = 20–58 years) selected from our normal MRI-SPECT databases. Although the mean age of the control group was 6 years more than the mean age of the RNMO group, this difference did not have statistical significance (p = 0.17, Student´s t-test).

### Ethics Statement

Written informed consent was obtained from all participant subjects. The study was approved by the Ethics Committee of the Center for Neurological Restoration of Havana.

### Image Acquisitions, Transformations and SPM Preprocessing

#### Brain MRI

MRI imaging was carried out using a 1.5 Tesla Symphony scanner (Siemens, Erlangen, Germany) according to the standardized protocol for MS [25]. High-resolution 3D T1-weighted magnetization-prepared rapid-acquisition gradient echo (MPRAGE) sagittal sections were also included to perform SPM (repetition time = 1970 ms, echo time = 3.9 ms, number of excitations = 1, flip angle = 15°, slice thickness = 1 mm, field of view = 25 cm and matrix size = 256×256).

Before the SPM preprocessing step, a lesion mask from manually segmented lesions visible on T2-weighted images was created for the patients with minor non-specific T2/FLAIR lesions. Manual lesion segmentation was performed using MRIcro software (www.sph.sc.edu/comd/rorden/mricro.html). The lesion mask was coregistered to the MPRAGE native space, using the rigid transformation calculated between the T2-weighted and MPRAGE images. The coregistered lesion mask was used to zero out respective areas in the patients' MPRAGE images, in order to reduce the influence of these lesions during segmentation and normalization procedures. These lesion areas were not incorporated into statistical analyses since they were few, small and present only in five patients. Although these lesion areas predominated in patients with more ON attacks (one patient with two, three patients with three, and one patient with four attacks), they were localized in different brain regions in each patient, which precluded potential bias towards patients with lesions for voxel-based analyses, considering also that we controlled for total intracranial volume. Furthermore, according to our data these lesions were negligible because there were no significant differences between RNMO and control groups for global tissue volumes (see results), even though eliminating these lesion areas might have created a bias towards positive results, especially for global WM volume.

The preprocessing step for SPM has recently been improved with the Diffeomorphic Anatomical Registration using Exponentiated Lie Algebra (DARTEL) registration method [26], which is implemented in the SPM8 software version used in this study (http://www.fil. ion.ucl.ac.uk/spm). First, we manually set the anatomical image origin at the anterior commissure and the orientation approximated Montreal Neurological Institute (MNI) space. Secondly, images were segmented into WM and GM images (bias corrected) using the standard unified segmentation model in SPM8. Thirdly, images were imported into a DARTEL form. Fourthly, the parameters of segmented images were used to generate custom DARTEL templates of all subjects. Lastly, WM and GM images were normalized to the MNI space using the DARTEL procedure to obtain warped and smoothed (12 mm-kernel) Jacobian modulated WM and GM images (voxel size = 1.5×1.5×1.5 mm^3^). Spatially normalized images were modulated in order to preserve the total amount of signal in the images [27]. WM/GM values for modulated images are typically referred to as GM/WM volume images (WMV/GMV images). We chose a 12 mm-kernel for smoothing in order to make our results comparable with other NMO SPM studies recently published that used the same kernel [12], [13]. Global tissue volumes (GM, WM and CSF) were estimated in the native space using the VBM8 toolbox (http://dbm.neuro.uni--jena.de/vbm8/).

#### Perfusion SPECT

SPECT imaging was performed with a double-head system (DST Xli, Sopha Medical Vision, France) equipped with ultra-high-resolution fan-beam collimators. The measured tomographic resolution for ^99m^Tc was 8.5 mm in the center of the image at a fixed radius of rotation of 150 mm. Image acquisition was started 10 min after injection of 555 MBq of 99mTc-ethyl cysteinate dimer into the antecubital vein of the right arm under resting condition (supine, eyes open, dimly lit, quiet room). Projection data were obtained in a 128×128 format for 64 angles at 26 s per angle. Total counts were equal or greater than 5×10^6^. A Butterworth filter was used for image back-projection reconstruction of SPECT images (cutoff frequency = 0.026 cycle/cm and order = 7). Attenuation correction was performed using Chang’s method [28] with attenuation coefficient µ = 0.085 cm.

SPECT images were corrected for partial volume effect (PVE) using the modified Müller-Gartner voxel-based method [29], which corrects also for the WM activity, in order to have genuine perfusion measurement by removing the effect of perfusion underestimation due to brain atrophy. All preprocessing steps for PVE correction were performed using the 2010-version of the ‘PVE-lab’ software (http://pveout.area.na.cnr.it). Each PVE-corrected SPECT image was then coregistered onto its respective native GM segment using the normalized mutual information procedure with trilinear Interpolation by SPM8. Coregistered SPECT images were normalized to MNI space by reapplying the same DARTEL parameters obtained previously for MRI during the SPM preprocessing step, including the 12 mm smoothing kernel. The use of the same DARTEL normalization parameters avoided differences due to the normalization process and also improved SPECT image alignment. DARTEL optimizes sensitivity of SPM by a more accurate alignment among the brains of different subjects [30], [31].

The resulting SPECT images were then scaled by the mean value per voxel of global GM counts, excluding counts from the cerebellum and occipital regions. This mean value was automatically extracted using the Marsbar toolbox (http://www.mrc-cbu.cam.ac.uk) and the anatomical labeling template [32]. GM counts from cerebellum and occipital regions were excluded because an exploratory study showed a decrease of relative perfusion (using global GM counts as reference value) with the increase of the number of ON attacks in the patient group, which mainly involved regions from the cerebellum and the occipital cortex. Thus, perfusion was measured in relative units using a reference value where perfusion was not affected by the attack-related process.

SPECT images were also masked by a binary image to exclude CSF space. The binary mask was created by summing up the WM and GM masks, which were obtained by thresholding WM and GM normalized mean images (of the entire sample) above a value of 0.25, corresponding to a higher than 25 percent chance for the voxel to belong to WM or GM, respectively. The mask was applied twice (before and after smoothing) to avoid contamination of misclassified voxels by smoothing in the first case and big edge effects in the second case.

The quality of the DARTEL normalization procedure was tested for the three image modalities by checking sample homogeneity using covariance as it is implemented in the VBM8 toolbox. SPECT images were tested before masking. For the three image modalities, volumes with overall covariance below two standard deviations were not observed, indicating that no outliers or volumes containing important artifacts were present. SPECT and MRI were carried out within a one-week interval at the most.

### Voxel-based Correlation Analysis

A separate multiple regression analysis in the RNMO group was carried out for the three image modalities using SPM8. Brain WMV, GMV or perfusion was considered as the dependent variable; while the number of ON attacks, LETM attacks, total attacks (ON plus LETM attacks), disease duration or NMO-IgG status (defined as a categorical variable: seronegative = 0; seropositive = 1) was considered as the independent variable (covariate of interest). Due to the high variability of the time interval from the last attack (see Table 1), this interval was added as another covariate of interest using the corresponding time interval (i.e. interval from last ON attack or last LETM attack). We also controlled for the standard nuisance covariates of age and sex for perfusion images and in the case of WMV/GMV images also total intracranial volume (TIV = GM+WM+CSF). T-contrasts were performed to test both positive and negative correlations with the covariate of interest. WMV and GMV images were masked by an absolute threshold of 0.25 to avoid contamination by misclassified voxels as much as possible. Since SPECT images were already masked, the absolute threshold was set to 0.

As supplementary analyses, we performed for each image modality a group-comparison between patients and control subjects using the standard nuisance covariates mentioned above. T-contrasts were performed in both positive and negative directions to map WMV/GMV and perfusion differences that were either increments or decrements in the RNMO group as compared with the control group.

All SPM analyses were performed using a statistical threshold of p<0.01(peak-level). The extent threshold used was determined by the cluster of voxels significant at p<0.05, corrected for multiple comparisons (family-wise error method) at the cluster level and after correction of non-isotropic smoothness. When a particular SPM analysis had a negative result, we explored this analysis using the same statistical threshold of p<0.01 but the extent threshold was determined by the expected number of voxels per cluster, which is estimated during SPM processing. The extent threshold, determined in this way, is a less restrictive control of false positives.

Anatomical regions were determined by comparing voxel and cluster location with a recently developed brain atlas [33]. This atlas contains 56 deep WM (DWM), 46 superficially located WM (SWM), 52 cortical GM, 10 subcortical GM and 10 other brain labels. The most significant voxels were reported in MNI coordinates.

### Global Tissue Volume Analyses

Similar to voxel-based analysis, we looked for association between global WM or GM volumes and the number of ON attacks, LETM attacks, total attacks, disease duration or NMO-IgG status, after controlling for age, sex and TIV by multiple regression analysis. Global WM and GM volumes of RNMO and control groups were also compared by the Student´s t-test. The significance level was set to 0.05. These analyses were carried out by STATISTICA software (Stat Soft, Inc, version 8.0).

## Results

In this study, by voxel-based correlation analysis, we aimed mainly to investigate possible associations between brain tissue volumes and perfusion and the attack-related process in patients with RNMO. Among analyzed covariates of interest (including the disease duration and NMO-IgG status), only the number of ON attacks showed significant correlation with regional brain tissue volumes (WMV and GMV) and perfusion. The time interval from the last ON attack did not show significant effect. For that reason, we repeated SPM correlation analyses using only the number of ON attacks as a covariate of interest to avoid unnecessarily reducing the statistical power, which did not change results. Correlations of regional brain WMV, GMV and perfusion with the number of ON attacks are described in the next subsections.

The number of ON attacks per patient (simultaneous or not with the LETM attack) was as follows: four patients with one attack; five patients with two attacks (one was blind in the left eye); five patients with three attacks (four were blind: two in the right eye, one in the left eye and one in both); and one patient with four attacks (with bilateral blindness). Table 1 summarizes the main demographical and clinical findings.

### Regional White Matter Volume Correlation with the Number of Optic Neuritis (ON) Attacks

**Figure 1:**
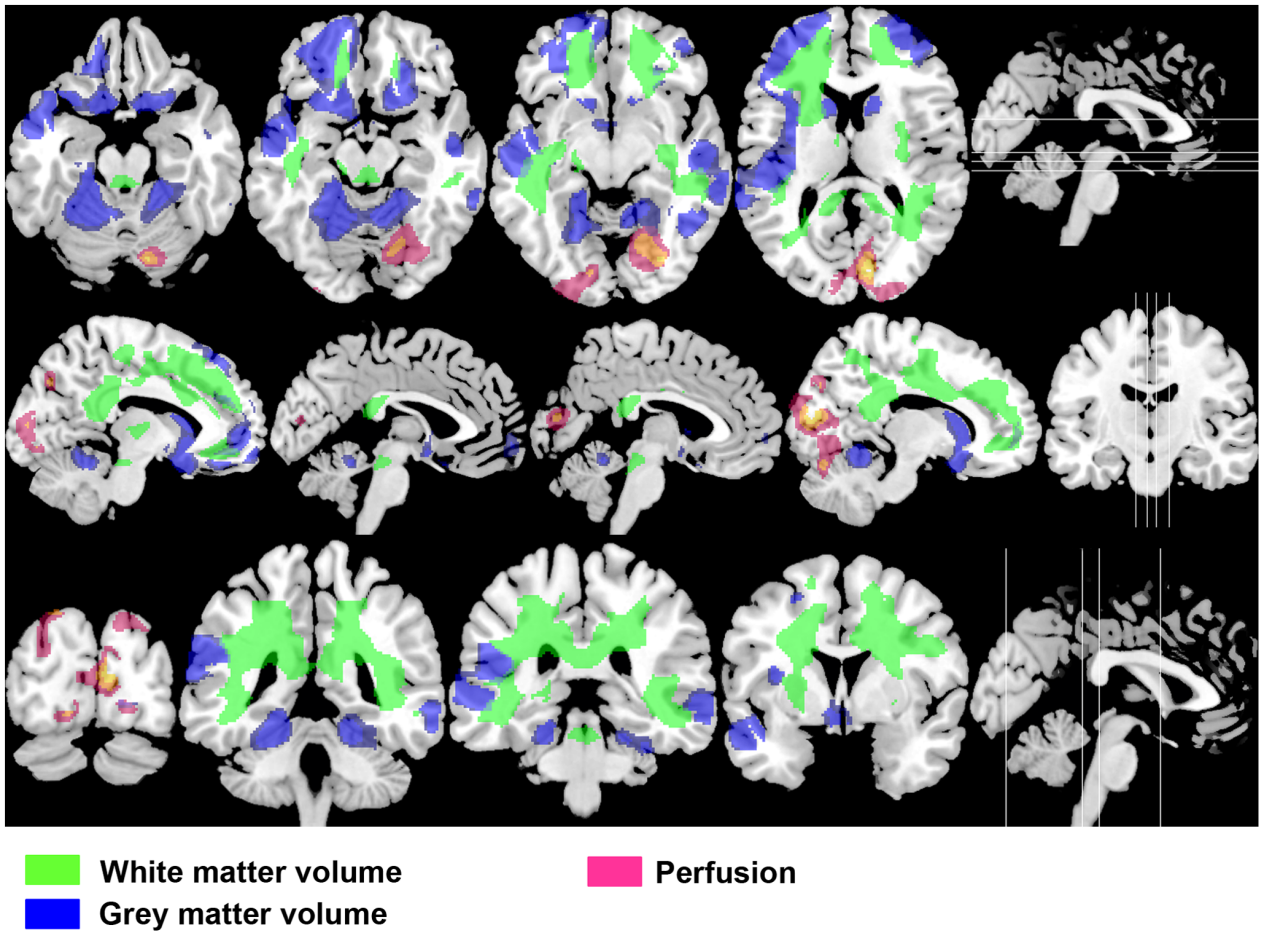
Multimodal negative correlation pattern with the number of optic neuritis attacks in RNMO patients. Axial, sagittal and coronal selected slices at four brain levels (levels shown as white lines in the figures on the right side), presenting overlays of negative correlation statistical parametric maps of white matter volume (in green), grey matter volume (in blue) and perfusion (in pink) on a T1-weighted high resolution MRI template. Decreases of tissue volumes and perfusion with the increase of the number of optic neuritis attacks comprised extensive brain regions, including important components of the visual system such as posterior thalamic radiations (white matter volume), middle and superior temporal gyri (grey matter volume) and the primary visual area (perfusion). Also note that tissue volume decreases included regions close to periaqueductal areas. Statistical parametric maps were thresholded at p value <0.01 (p value <0.05 corrected for multiple comparisons at the cluster level).

A significant decrease of regional WMV as the number of ON attacks increases (i.e. negative correlation) was found in one extensive cluster that comprised DWM and SWM bilateral regions (Table 2 and Figure 1 in green). Note that significant regions included areas close to the lateral and fourth ventricles. The most significant voxel in this cluster was found at the left superior longitudinal fasciculi (MNI: x, y, z = −28, −45, 32; P<10^−3^, T = 8.91). Significant increases were not found for any region, as the number of ON attacks increased (i.e. positive correlation).

**Table 2:** Brain regions with negative correlation between white matter volume and the number of optic neuritis attacks.

P	No. of voxels	Brain regions
10^−3^	83360	**Bilateral DWM:** superior longitudinal fasciculus; superior and inferior fronto-occipital fasciculus; posterior thalamic radiation; anterior, posterior and superior corona radiata; cingulum (cingulated gyrus); cingulum (hippocampus); fornix(cres) stria terminalis; sagittal stratum; external capsule; anterior limb, posterior limb and retrolenticular part of internal capsule; genu, body and splenium of corpus callosum; tapatum; midbrain; superior cerebellar peduncle; and the pons. **Left DWM:** cerebral peduncle. **Bilateral SWM:** superior parietal; postcentral; angular; supramarginal; cingulum; precuneus; cuneus; middle and inferior occipital; superior, middle and inferior temporal; precentral; middle and inferior frontal; lateral and middle fronto-orbital; and rectus. **Left SWM:** superior occipital and fusiform.

P, P value corrected for multiple comparisons at cluster level; DWM, deep white matter; SWM, superficially located white matter.

### Regional Grey Matter Volume Correlation with the Number of ON Attacks

Like WMV, a significant negative correlation between regional GMV and the number of ON attacks was found in two clusters. The first cluster comprised regions of the left temporal and parietal lobes and other brain regions bilaterally as shown in Table 3 and Figure 1 (in blue), including areas close to the third ventricle. The most significant voxel in this cluster was found at the left superior temporal gyrus (MNI coordinates: x, y, z = −43, −23, 3; P<10^−3^, T = 8.03, Brodmann area {BA} 48). The second cluster involved the right temporal lobe and other areas on both cerebral and cerebellar hemispheres, including regions close to the fourth ventricle. The most significant voxel in this cluster was found at the right lingual gyrus (MNI: x, y, z = 24, −55, 2; P<10^−3^, T = 4.43, BA 19). No significant positive correlation was observed.

**Table 3:** Brain regions with negative correlation between grey matter volume and the number of optic neuritis attacks.

P	No. of voxels	Brain regions
0.004	46077	**Left GM:** superior and inferior temporal gyri; hippocampus and parahippocampal gyrus; cingulated, precuneus, postcentral, angular and supramarginal gyri. **Bilateral GM:** insular; caudate nucleus; putamen; superior, middle and inferior frontal gyri; lateral fronto-orbital and middle fronto-orbital gyri; and the rectus gyrus.
0.041	15955	**RightGM:** superior, middle and inferior temporal gyri. **Bilateral GM:** lingual and fusiform gyri; and the cerebellum.

P, P value corrected for multiple comparisons at cluster level; GM, grey matter.

### Regional Brain Perfusion Correlation with the Number of ON Attacks

Similar to WMV and GMV, a significant negative correlation was found in one cluster, mainly located in the occipital cortex bilaterally, including the primary visual area and the right cerebellum (Table 4 and Figure 1 in pink). Although this cluster was mostly situated on GM, adjacent WM regions were also observed. The most significant voxel in this cluster was found at the right cuneus (MNI: x, y, z = 11, −83, 12; P<10^−3^, T = 5.75, BA 17).

**Table 4:** Brain regions with negative correlation between perfusion and the number of optic neuritis attacks.

P	No. of voxels	Brain regions
0.017	10801	**Bilateral GM:** cuneus and lingual gyri; superior, middle and inferior occipital gyri; fusiform and cingulated gyri. **Right GM**: cerebellum. **Left GM:** supramarginal and angular gyri. **Bilateral SWM:** cuneus; superior, middle and inferior occipital. **Right SWM:** lingual and fusiform. **Left SWM:** superior parietal.

P, P value corrected for multiple comparisons at cluster level; GM, grey matter; SWM, superficially located white matter.

**Figure 2:**
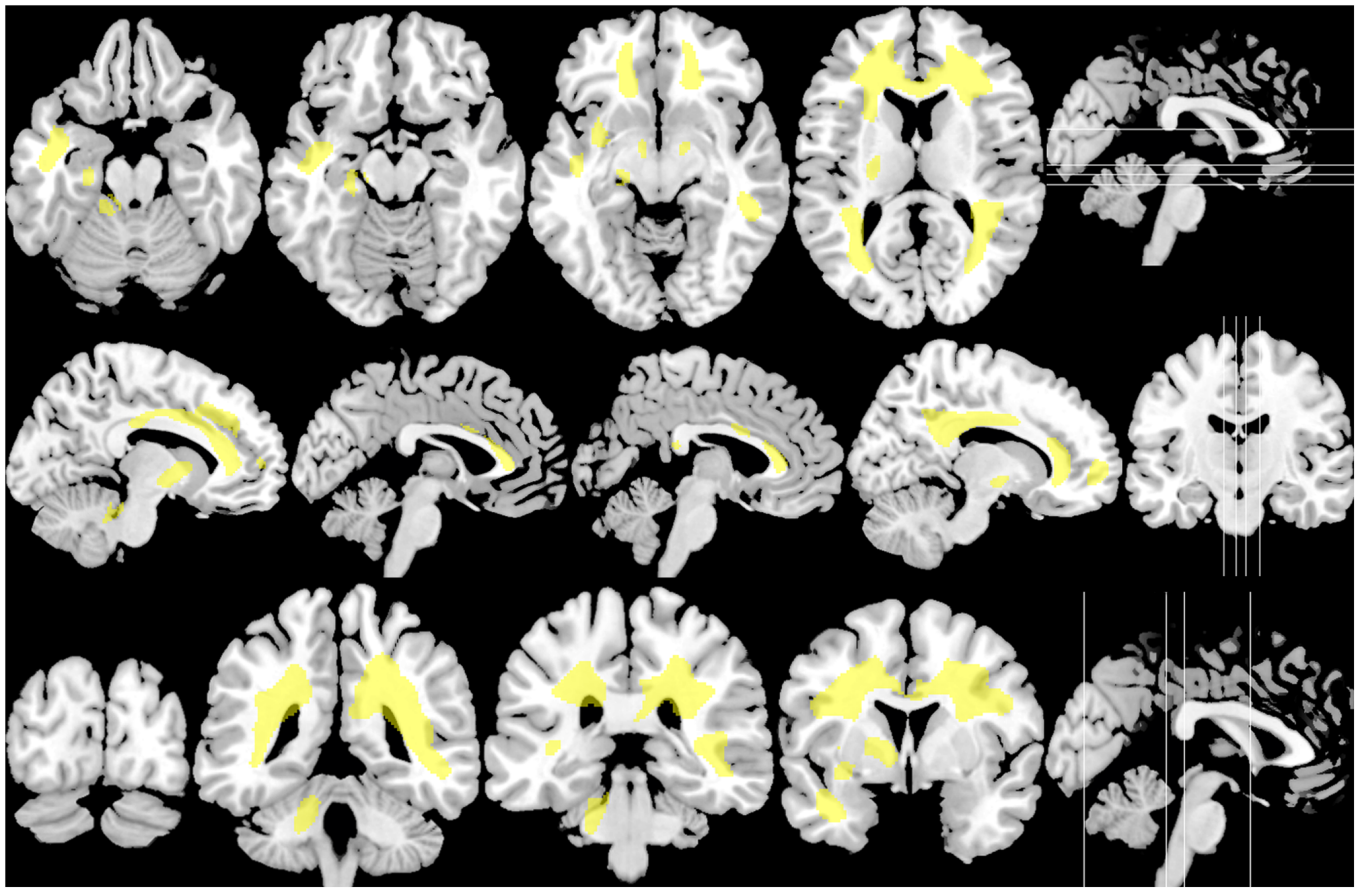
Brain perfusion positive correlation with the number of optic neuritis attacks in RNMO patients. The same slices as shown in Figure 1, presenting the perfusion positive correlation statistical parametric map, which was found in an extensive brain area mainly located in the white matter. Note that perfusion changes included periventricular areas. Statistical parametric map was thresholded at p value <0.01 (p value <0.05 corrected for multiple comparisons at the cluster level).

**Figure 3:**
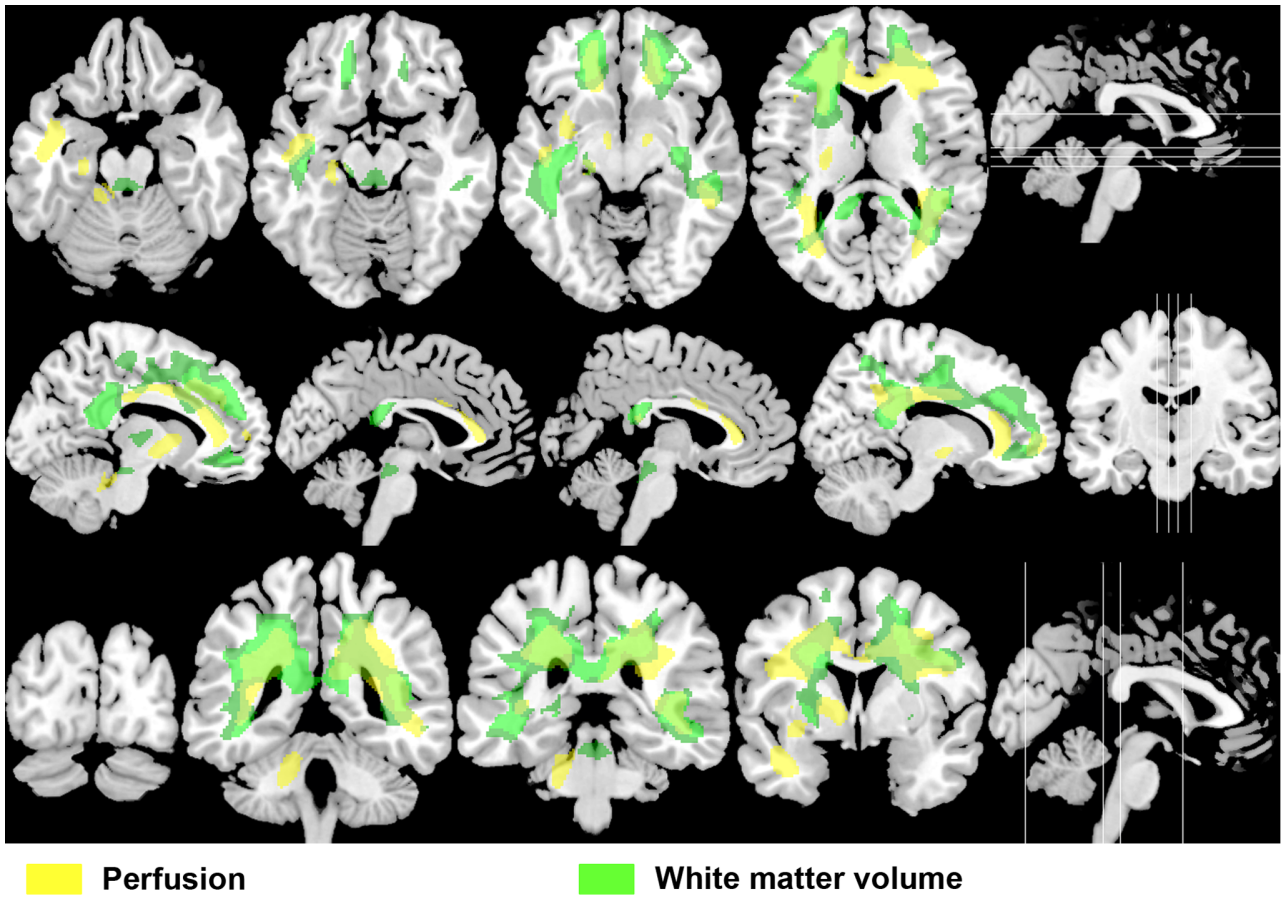
Overlapping perfusion and white matter volume changes in opposite directions. The same slices shown in Figures 1 and 2, presenting positive perfusion correlation statistical parametric map (in yellow), mostly overlapping the brain area where white matter volume negatively correlated with the number of optic neuritis attacks (in green). This suggests that perfusion alteration could be important in the development of brain structural abnormalities in RNMO.

Contrary to WMV and GMV, a significant perfusion positive correlation was also found in one extensive cluster as shown in Table 5 and Figure 2 (in yellow), mostly overlapping the WM area where WMV showed opposite changes (negative correlation) with the number of ON attacks (Figure 3). This cluster included DWM and SWM bilateral regions, the cerebellar peduncle (DWM) and the cerebellum (SWM) on the left side. Note that significant regions involved areas close to the lateral, third and fourth ventricles. Although the cluster was mainly located on WM, it also comprised adjacent GM regions, including the left cerebellar hemisphere. The most significant voxel in this cluster was found at the left anterior corona radiata (MNI: x, y, z = −15, 32, 24; P<10^−3^, T = 8.60).

**Table 5:** Brain regions with positive correlation between perfusion and the number of optic neuritis attacks.

P	No. of voxels	Brain regions
10^−3^	63648	**Bilateral DWM:** superior longitudinal fasciculus; superior and inferior fronto-occipital fasciculus; posterior thalamic radiation; anterior, posterior and superior corona radiata; cingulum (cingulated gyrus); cingulum (hippocampus); fornix(cres) stria terminalis; sagittal stratum; external capsule; anterior limb, posterior limb and retrolenticular part of internal capsule; genu, body and splenium of corpus callosum; tapatum; cerebral peduncle; and substancia nigra. **Left DWM:** superior and inferior cerebellar peduncle. **Bilateral SWM:** superior parietal; postcentral; angular; supramarginal; cingulum; precuneus; cuneus; superior and middle occipital; superior, middle and inferior temporal; precentral; superior, middle and inferior frontal; lateral fronto-orbital; and rectus. **Right SWM:** inferior occipital. **Left SWM:** cerebellum. **Bilateral GM:** cingulate gyrus; superior, middle and inferior frontal gyri. **Left GM:** parahippocampal gyrus; superior temporal gyrus; insular; hippocampus and the cerebellum.

P, P value corrected for multiple comparisons at cluster level; DWM, deep white matter; SWM, superficially located white matter; GM, grey matter.

### Supplementary Analysis

No significant differences were found between RNMO patients and controls for the three image modalities. Nevertheless, when the same analyses were explored using the extent threshold determined by the expected number of voxels per cluster, only brain perfusion showed significant decrease in the RNMO group as compared with the control group. The perfusion decrease comprised an extensive brain area, mostly involving regions that showed both negative and positive correlation with the number of ON attacks (Figure S1).

### Global Tissue Volumes

We only found significant negative correlation between the global WM volume and the number of ON attacks (P = 0.014; Beta = −0.28; and 95% confidence interval between −0.49 and −0.07). There was no significant correlation between the global GM volume and the number of ON attacks (P = 0.06; Beta = −0.29; and 95% confidence interval between −0.61 and 0.02). There were no significant differences between RNMO and control groups for the global WM volume (NMO = 394.05±58.68 ml; Control = 434.49±57.72 ml; P = 0.07) and for the global GM volume (NMO = 609.38±59.23 ml; Control = 618.02±63.53 ml; P = 0.7).

## Discussion

Voxel-based correlation analysis with the number of ON attacks as covariate of interest allowed us to find a subtle and common effect at the group level for each neuroimage modality analyzed, revealing an underlying ON attack-related process in RNMO patients. A multimodal negative correlation pattern with the number of ON attacks was found, which comprised regional brain WMV, GMV and perfusion. Regional WMV changes were reflected in a significant decrease in the global WM volume with the increase in the number of ON attacks. We also found a positive regional correlation pattern of brain perfusion with the number of ON attacks, mostly overlapping the WM area where regional WMV showed opposite changes. These observations are discussed below.

The wide extent of WMV changes observed is congruent with a recent study by Rueda Lopes and coworkers using for the first time a voxel-based approach to analyze DT MRI in NMO [9]. This study identified extensive WM damage in the NAWM in patients with NMO. Their findings were similar to our results and more extensive than previously reported by DT MRI using a region-of-interest–based analysis [8], [10], [11], which is observer-dependent and hypothesis driven, in contrast to voxel-based approach. Rueda Lopes and coworkers showed that damages were present in WM regions connected to the optic nerves and spinal cord, as initially reported [8], [10], [11], but also in not connected WM regions. This study also explored whether the number of clinical relapses (equivalent to total number of attacks) was correlated with DT imaging parameters and found no significant correlation [9], in agreement with our findings. The fact that we did not find significant correlation with the number of LETM attacks either, suggests that the LETM-attack effect on the brain is weaker in comparison with ON attacks. Consequently, the use of the total number of attacks for correlation analysis might be obscuring the specific correlation with the number of ON attacks.

A possible explanation to a subtle and extensive tissue volume decrement (including GMV), could be that there is a direct relationship between damage to the anterior visual pathway (especially in the optic nerve) and global brain atrophy in RNMO as shown in MS [34]–[36], resulting from a progressive diffuse brain degenerative process beyond the CNS demyelinating component. Although possible, this does not account for our results (directly related to ON attacks) because this association has been observed in patients with [34] and without previous history of ON events [35]. A recent study also showed that this relationship is abolished for GMV in patients with history of ON, remaining only for WMV independent of ON attacks [36].

On the other hand, the multimodal changes observed were inter-related since they were defined by the number of ON attacks. Recent studies demonstrating the lack of cortical pathology in NMO [37]–[39] suggest that GMV changes are secondary to WMV changes, in agreement with the noticeable correspondence of WMV changes with adjacent GMV changes as shown in Figure 1. GMV changes might be associated to microstructural damage due to retrograde axonal degeneration with impact on cortical neurons, not detectable even at 7-Tesla MRI as has been recently suggested by Sinnecker and colleagues (38), which is coherent with previous fMRI, MT MRI and DT MRI studies showing abnormalities in the NAGM in NMO patients [8], [14]–[16]. GMV changes included the bilateral middle and superior temporal gyri, which are both part of the dorsal stream of the visual system devoted to spatial tasks [40].

The fact that perfusion decrease in the occipital cortex, including the primary visual area, with the increase in ON attack number was not accompanied by local GMV changes indicates that this decrease is also secondary to WMV changes. WMV changes involved tracts directly connected with the occipital cortex (e.g. posterior thalamic radiations or optic radiations), which suggests that this perfusion decrease is a diaschisis phenomenon as a result of anterograde degeneration in connected tracts.

Thus our study shows for the first time that the increase in ON attack number in patients with RNMO, which is directly related with incremental visual disability, decreases regional brain tissue volumes and perfusion bilaterally, involving important components of the posterior visual system such as the posterior thalamic radiations (WMV), middle/superior temporal gyri (GMV) and the primary visual area (perfusion). This is consistent with clinical findings that blindness was only present in one out of nine patients with less than three ON attacks, while five out of six patients with three or four ON attacks were blind.

These data fit well with the growing recognition of specific transsynaptic degeneration (antero- or retrograde) in the visual system in different neurological conditions directly affecting part of the visual pathway [41]–[45]. It is plausible that ON attacks in RNMO also have damaging impact on other parts of the visual system as distant as the primary visual area, as has been recently suggested by Pfueller and coworkers in MS patients [44]. Previous DT MRI studies in NMO uphold this idea as well [8], [10], [11]. However, transsynaptic degeneration would only explain our findings partially bearing in mind that ON-attack related changes involved other regions not belonging to the visual pathway.

Considering the extension of tissue volume changes, they possibly lead also to cognitive dysfunction. Recently, it has been reported that NMO patients with cognitive impairment have extensive WM atrophy compared to NMO patients without it [12]. We could not assess the interaction of the ON-attack related process and cognitive function because none of our patients had clinical signs of cognitive dysfunction at the time of evaluation, which not necessarily signifies that cognitive dysfunction was absent in our patients since cognitive function was not one of our aims and thus sophisticated cognitive tests were not applied.

It is noteworthy as well that the decrease of tissue volume with the number of ON attacks included regions that are close to periaqueductal areas, which would explain the radiological observation that in NMO patients, especially in late stages, standard brain MRI reveals T2-weighted lesions in these regions [46].

Unexpectedly, brain perfusion changes comprised regions where perfusion increases with the number of ON attacks, involving an extensive WM area. These changes seem to have a different origin from perfusion changes observed in the occipital cortex. They are also different to those observed in MS patients (reviewed in [47] and [48]) and could be associated to the complex and not yet fully understood pathophysiology of NMO [1].

WM perfusion changes could be due to increased perfusion with regard to normal values or perfusion restoration starting from lowers than normal values. The fact that group-comparison analysis also showed perfusion decrement in the RNMO group compared with the control group, in similar WM regions (Figure S1), upholds that patients during the early stages of the ON attack-related process have WM hypoperfusion, which eventually decreases in later stages. This provides evidence that CNS vasculature is an early target of the disease process, which agrees with immunopathological studies [19]–[21].

Early WM hypoperfusion is possibly related to early vascular impairment and the perivascular astrocyte destruction observed in NMO [19]–[21], [49]. Recent evidence supports that these processes could be associated to NMO IgG that targets the aquaporin-4 (AQP4) water channel protein (reviewed in [2]), which is found in perimicrovessel astrocyte foot processes, glia limitans and ependyma [50]. Since we didn’t find association of NMO-IgG status with brain perfusion (nor tissue volumes) we can assume three possibilities: 1) our findings are independent of NMO-IgG status; 2) the expected effect is weak and to detect it a larger sample size is needed; and 3) all or some of seronegative patients were actually false negatives and we could not detect them because of the sensitivity of the assay used [51] or NMO-IgG activity is reduced during the stable phase of the disease [52]. Further studies using more sensitive and specific second generation techniques may be needed to clarify the association between ON attack-related WM perfusion changes and NMO-IgG status. However, it is interesting that WM perfusion changes included periventricular areas, as illustrated in Figure 2, corresponding to brain regions of high AQP4 expression [50], [53].

Even more surprising is the fact that WM perfusion and WMV correlations have opposite signs and overlap extensively as shown in Figure 3. It is possible to consider that ON attack-related WM perfusion increase is an artifact induced by PVE correction, since it implies dividing SPECT images by MPRAGE images. We explored this possibility by repeating the same correlation analysis using non PVE-corrected SPECT images (where perfusion is underestimated due to atrophy) and we found comparable results (data not shown), which confirmed that our finding is not a PVE-related artifact. We decided to use PVE-corrected images because it improves the sensitivity of the voxel-based analysis approach [54]. Even after PVE correction, which removes perfusion underestimation, a direct relationship is expected between WMV and WM perfusion changes since both processes have in common that they reflect decrease in metabolic activity. However, it seems that additional processes take place, enhancing the perfusion/metabolic counterpart of WMV loss. These additional processes could be connected with reparative astrogliosis effected by a population of astrocyte progenitor cells, as recently observed in NMO lesions, which has not previously been described in other demyelinating diseases [49].

On the other hand, the fact that ON attack-related changes were not affected by the time elapsed from the last attack, suggests that WM hypoperfusion after the initial attack persists for some time during the stable phase of the disease. It is conceivable that early WM hypoperfusion might lead to metabolic dysfunction in the WM tissue and be responsible in part for cellular damage to astrocytes and gradual demyelination as a result of secondary injury to oligodendrocytes, which raises the supposition that it could be important in the development of brain structural abnormalities in RNMO, especially in WM. This interpretation is in line with the voxel-based DT MRI study of Rueda Lopes and coworkers [9], demonstrating that damages in NAWM in NMO were not only related to axonal degeneration (axial diffusivity decrease) secondary to lesions in the optic nerves and spinal cord, but also to demyelination (radial diffusivity increase) as a result of damage to oligodendrocytes, which is corroborated in histopathological studies [19], [55].

Taken together, our results point to ON attacks being only the tip of the iceberg and that our brain image findings detect an occult subclinical and more extensive part of a subtle pathological process that evolves with each new ON attack. This process is not associated to disease duration as we didn’t find correlation between this clinical variable and brain tissue volumes and perfusion. Disease duration is not related to the number of attacks. ON and LETM attacks (in discrete attack or simultaneously) can be separated by weeks to years in RNMO [18]. Instead, our findings seem to be associated with early and extensive WM hypoperfusion and the subsequent pathological process apparently related to this initial event, in which only the ON attacks (and related symptoms) are clinically detectable.

To better elucidate the role of early WM hypoperfusion as a pathogenetic mechanism in this disease, more studies are required. A longitudinal evaluation of RNMO patients with confirmed NMO-IgG status (or equivalent animal model) that includes both acute and stable phases of the disease and a multimodal neuroimaging approach using advanced MRI and PET imaging may be useful for such studies. This would allow tracing the spatial and temporal hemodynamic, metabolic, molecular and structural changes behind the attack-related process. As far as we know, PET imaging has never been used to investigate NMO.

This study has limitations. First, it is based on a small number of patients, although it is substantial considering the low incidence of NMO. Still, our main findings seem to be robust since RNMO is relatively homogeneous with regard to the clinical feature that the incremental visual disability is ON attack-related, independent of geographic regions, ethnic groups or other demographic variables. Therefore, similar results should be expected in other populations of RNMO patients. Second, in spite of using recently improved techniques for spatial segmentation, coregistration and normalization; our results should be interpreted with care because we cannot exclude possible caveats associated to the SPM preprocessing step, especially for WMV images. Third, perfusion measurement was made in relative units. Yet, there are no reasons to presume that absolute perfusion measurement based on MRI or CT techniques would find different results. Four, although our data suggest a role for NMO IgG, we were not able to associate our main findings to NMO-IgG due to limited sensitivity of the assay used to detect seropositivity status. Finally, this study is cross-sectional and should be replicated in a longitudinal study including both acute relapse and stable phases of the disease in patients with confirmed NMO-IgG status. Considering the uncommonness of NMO, a multicenter study would be a more appropriate approach.

### Conclusions

By means of voxel-based correlation analysis, our study demonstrates that regional tissue volumes (WMV and GMV) and perfusion gradually decrease as the number of ON attacks increases in patients with RNMO, involving important components of the visual system. This could be of relevance to the comprehension of incremental visual disability during the disease course. We also found that perfusion increases with the number of ON attacks in an extensive WM area, starting from lowers than normal values; and mostly overlapping the region where WMV decreases in association to the ON attack-related process. This provides evidence that CNS blood microvessels are early targets of the disease and suggests that early WM hypoperfusion could be important in the development of brain structural abnormalities in RNMO.

## Supporting Information

Figure S1 **Axial, sagittal and coronal selected slices at four brain levels (levels shown as white lines in the figures on the right side) presenting the statistical parametric map of brain perfusion decrease in the RNMO group compared with the control group (in cyan).** This group-comparison analysis was performed using an extent threshold determined by the expected number of voxels per cluster, which is a less restrictive control of false positives. Negative (in pink) and positive (in yellow) correlation statistical parametric maps of perfusion with the number of optic neuritis attacks are also shown as presented in Figures 1 and 2, respectively. The perfusion decrease comprised an extensive brain area, mostly involving regions that showed both negative and positive correlation with the number of ON attacks.
(TIF)
