# Supplementary figures and images for: Brain Tissue Volumes and Perfusion Change with the Number of Optic Neuritis Attacks in Relapsing Neuromyelitis Optica: A Voxel-Based Correlation Study
Source: PLoS One. 2013 Jun 18;8(6):e66271. doi: 10.1371/journal.pone.0066271 (PMC3688888; doi:10.1371/journal.pone.0066271)

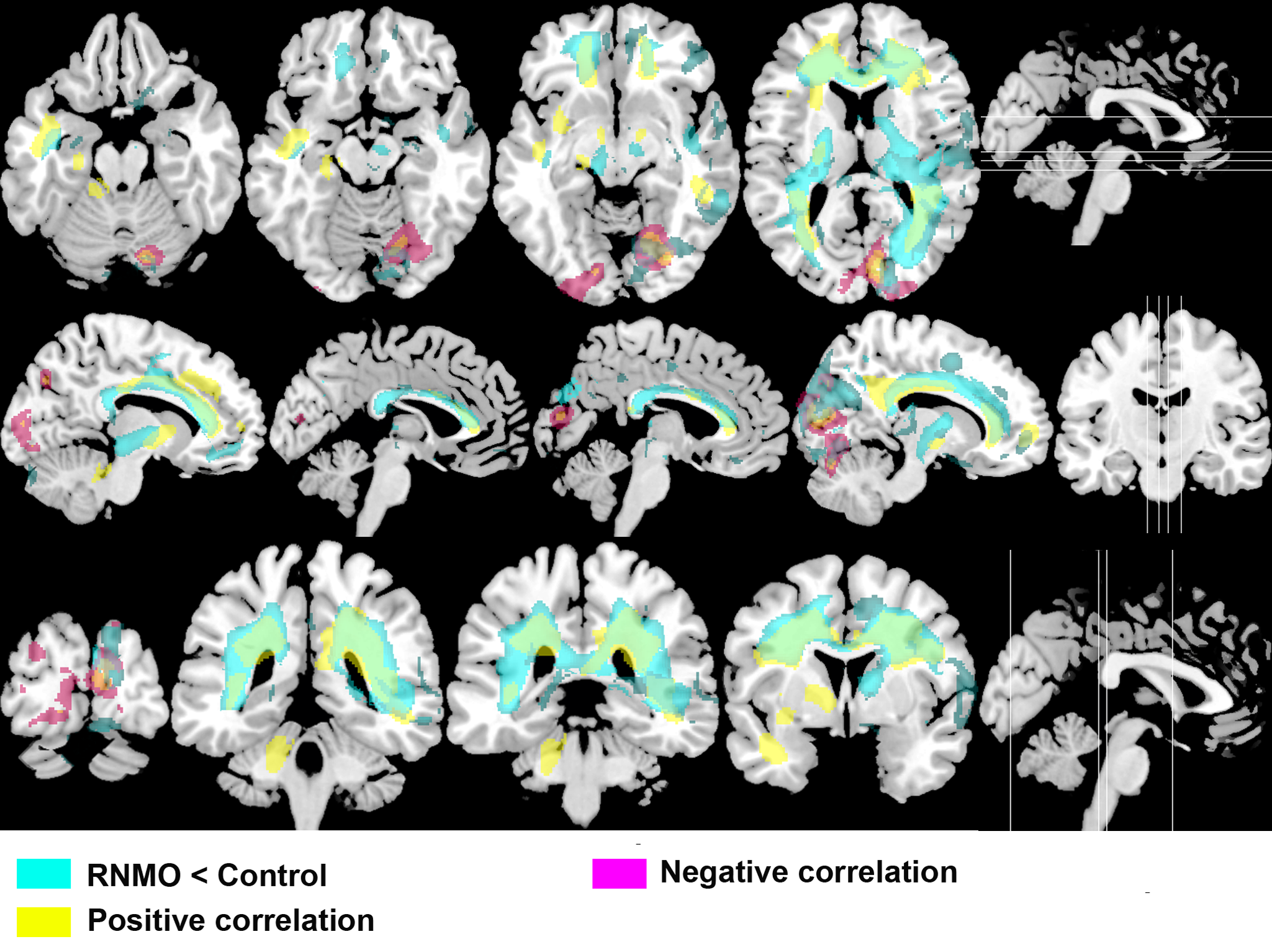

Supplement: Figure S1 — Axial, sagittal and coronal selected slices at four brain levels (levels shown as white lines in the figures on the right side) presenting the statistical parametric map of brain perfusion decrease in the RNMO group compared with the control group (in cyan). This group-comparison analysis was performed using an extent threshold determined by the expected number of voxels per cluster, which is a less restrictive control of false positives. Negative (in pink) and positive (in yellow) correlation statistical parametric maps of perfusion with the number of optic neuritis attacks are also shown as presented in Figures 1 and 2, respectively. The perfusion decrease comprised an extensive brain area, mostly involving regions that showed both negative and positive correlation with the number of ON attacks. (TIF) [file pone.0066271.s001.tif]
